# Management of aplastic anaemia in pregnancy in a resource poor centre

**DOI:** 10.11604/pamj.2016.24.277.9880

**Published:** 2016-07-28

**Authors:** Celestine Osita John, Kaladada Korubo, Rosemary Ogu, Chigozirim Faith Mmom, Alpheus Gogo Mba, Ezenwa-Ahanene Chidiadi, Chris Akani

**Affiliations:** 1 Department of Obstetrics and Gynaecology, University of Port Harcourt Teaching Hospital, Port Harcourt, Nigeria; 2 Department of Haematology & Blood Transfusion, University of Port Harcourt Teaching Hospital, Port Harcourt, Nigeria

**Keywords:** Aplastic anaemia, pregnancy, transfusion, bone marrow, cyclosporine, platelet concentrate, absolute neutrophil count

## Abstract

Aplastic anaemia occurring in pregnancy is a rare event with life threatening challenges for both mother and child. We present a successful fetomaternal outcome despite the challenges in the management of this rare condition in a tertiary but resource poor centre. This is case of a 37 year old Nigerian woman G6P0^+5^managed with repeated blood transfusions from 28 weeks of gestation for bone marrow biopsy confirmed aplastic anaemia following presentation with weakness and gingival bleeds. She had a cesarean section at 37 weeks for pre-eclampsia and oligohydraminous with good feto-maternal outcome. She was managed entirely with fresh whole blood and received 21 units. Aplastic Anaemia in Pregnancy is a rare event with poor feto maternal prognosis. Successful management is possible with good multi-disciplinary approach and availability of supportive comprehensive obstetric care.

## Introduction

Aplastic anaemia (AA) is a rare bone marrow (BM) failure syndrome which occurs even more rarely in pregnancy. In AA, there is failure of production of haematopoietic stem cells and precursor cells associated with aplasia and fatty replacement of the marrow. Subsequently, there is depression of all the cell lines with attendant anaemia, neutropoenia and thrombocytopoenia (pancytopoenia) [1]. Patients with AA are predisposed to recurrent infections and may have a bleeding diathesis [1–3]. Its' management poses a lot of challenges to the obstetrician due to the rarity and poor prognosis of fetal and maternal outcome. Good multidisciplinary collaboration is key for successful outcome. There exist little documented cases or study of aplastic anaemia in pregnancy in Nigeria. This is the first documented case of aplastic anaemia in pregnancy in University of Port Harcourt Teaching hospital, in South-South Nigeria. We present the case, our challenges and management carried out to achieve a successful feto-maternal outcome.

## Patient and observation

**Figure 1:**
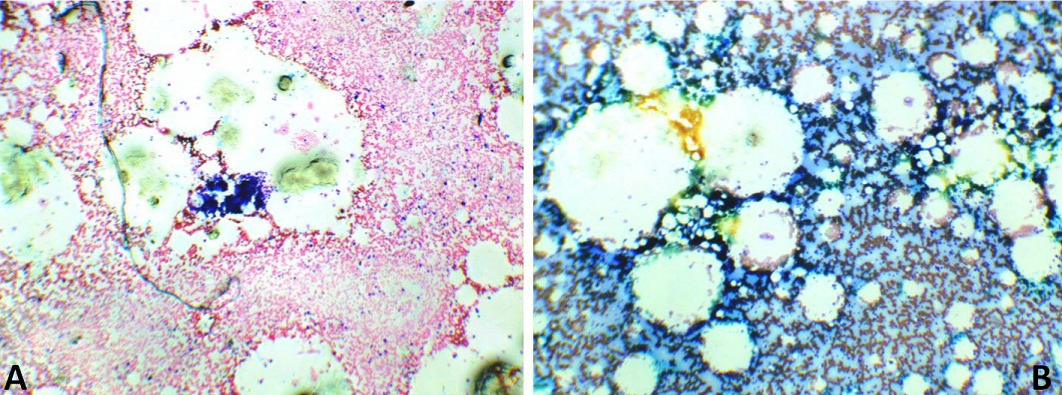
Bone marrow aspirate (A) and (B) showing hypocellularity, increased fat cells and reduced number of haemopoietic cells. There are no megakaryocytes and erythropoiesis is depressed

**Figure 2:**
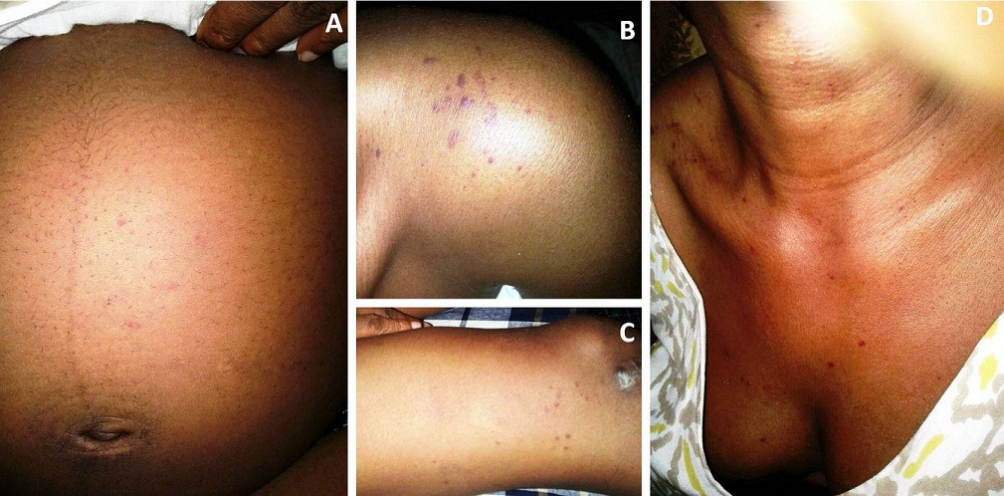
Purpura and petechiae haemorrhages on the abdomen (A), top left shoulder (B), right thigh (C), anterior chest wall and neck (D)

Mrs KA was a 37 year old Gravida 6 Para 0^+5^/ (G6P0^+5^/ ), who presented to the University of Port Harcourt Teaching Hospital (UPTH) on the 28th October, 2015 at 28 weeks gestation with history of weakness and occasional gingival bleeding of 2 months duration. She had never had these complaints prior to pregnancy. She had no history of jaundice, haematuria, bleeding from any other orifice or spontaneous bruising. There was no familial history of similar experience. She admitted taking some local oral herbal medications for secondary infertility of 3 years duration. She took this herbal medication for about 18 months and stopped one year before pregnancy was achieved spontaneously. The symptoms of weakness and gingival bleeding started at about 20 weeks of gestation. Prior to presentation at UPTH, she had received 5 units of whole blood transfusion in a private clinic over a period of four weeks. She was married to a 39 year old unemployed graduate and most of the financial aspect of her care was handled by her siblings. She came on self referral following her elder sisters' insistence. On examination at presentation, she was markedly pale, afebrile, anicteric and had no pedal edema. Her blood pressure was 120/70mmHg and pulse rate was 90 beats per minute. Her abdomen was gravidly enlarged with a symphisio-fundal height of 29cm and a singleton active fetus. She was reviewed with the haematology team, admitted, investigations commenced and placed on double dose haematinics and antimalarials. Her investigations at presentation revealed: low Haemoglobin (Hb) of 7g/dl, normal White Blood Cell (WBC) Count and Absolute Neutrophil Count (ANC) of 5.1 X10^9^//L and 2.6 X10^9^//L respectively with low Platelet Count of 07 X10^9^//L and a low Absolute Reticulocyte Count (ARC) 21 X 10^9^//L. Significant peripheral blood film findings were a normochromic, normocytic anaemia with severe thrombocytopoenia. There were no blasts neither was there dysplasia. Her blood group was B+, Hb genotype was HbAA and direct antiglobulin test was negative. Screening for HIV, hepatitis B and C were all negative. Her urinalysis was normal. Stool analysis showed no abnormality. Obstetric ultrasonography noted an active singleton fetus at 27 weeks. For unforeseen reasons, she was only able to get a bone marrow aspiration and biopsy done 2 weeks after admission. BM aspiration revealed a hypocellular (marrow cellularity 20%), fatty marrow with depressed erythropoiesis, granulopoiesis, megakaryopoiesis and no associated dysplasia or blasts (Figure 1). The BM biopsy confirmed the diagnosis of aplastic anaemia. She received 7 units of fresh whole blood transfusion over a period of 2 weeks. Her post transfusion Hb was 10g/dl, ANC was 2.2 X10^9^//L and platelet count was 8 X10^9^//L. She was placed on weekly fresh whole blood transfusion as platelet concentrate was unavailable. At 33 weeks gestation, she developed hypertension and was commenced on oral antihypertensive agents. However, following serial obstetric ultrasonography that noted moderate oligohydraminous and an estimated fetal weight of 2.6kg at 37weeks gestation, she had emergency caesarean section on 31/12/15. Thirty minutes prior to the surgery and also intra-operatively, she received more fresh whole blood transfusions. She was delivered of a live male neonate of birth weight 2.9kg, with Apgar scores of 8 and 9 in the first and fifth minute respectively. Estimated blood loss was 800mls. She was observed in the intensive care unit for 24 hours and later transferred to the postnatal ward. She made good clinical improvement on broad spectrum parenteral antibiotics, analgesics, haematinics and whole blood transfusions as platelet concentrate remained unavailable. Her Hb on the second post operative day was 13g/dl, WBC 3.7 X10^9^//L, ANC was 1.5 X10^9^//L while platelet count was 02 X10^9^//L. She was also noticed to have developed fresh purpura and petechial haemorrhages on her limbs, shoulder and trunk. (Figure 2). Due to unavailability of platelet concentrates, she received 2 more units of fresh whole blood. She was also commenced on Cyclosporine 300mg twice daily while arrangements were being made to secure platelet concentrate from another centre, as the anaemia had been corrected. Her clinical condition improved despite the severe thrombocytopoenia; gingival bleeding reduced and the petechial haemorrhages resolved. She was discharged home on the 10^th^/ day postpartum following patient and relatives' complaints of financial and psychological issues with continuous admission. Her Hb was 14.7g/dl, WBC 4.0 X10^9^//L, ANC 1.9 X10^9^//L while platelet count was 8 X10^9^//L. A repeat bone marrow biopsy was not done. She was to continue with weekly outpatient appointment to the haematology clinic. She however defaulted for eight weeks. She was seen thereafter and is presently on cyclosporine and encouraged to continue follow-up.

## Discussion

Aplastic anaemia is rare in pregnancy. It is caused by destruction of pluripotent stem cells in the bone marrow [1–3]. It has an annual incidence of 2-6/million, with the incidence higher in Asia about 2-3 fold than Europe [3]. Aplastic anaemia may be congenital or acquired. Acquired AA may be associated with paroxysmal nocturnal haemoglobinuria (PNH). Autoimmunity, viruses, drugs, chemicals, radiation or pregnancy, however acquired AA is most often idiopathic in up to 80% of cases [1, 2, 4]. The exact relationship between AA and pregnancy is unclear. In some patients, pre-existing AA is exacerbated by pregnancy and marrow function improved on termination of pregnancy [4]. Pregnancy on itself has been said to be a trigger factor as termination of pregnancy [4]. Some cases develop AA during pregnancy and it reoccurs in subsequent pregnancies [4]. termination of pregnancy in some patients led to remission of the disease in an otherwise healthy women [5]. Our patient was otherwise healthy until she became pregnant. However she took some local oral herbal medications whose chemical component cannot be ascertained. Clinically, patients with aplastic anaemia present with weakness, easy fatigueability, recurrent infections, mucosal bleeds and skin haemorrhages such as petechiae purpura and ecchymoses [1, 2, 6, 7]. Our patient had these symptoms of anaemia, bleeding gums, purpura and petechiae haemorrhages. Diagnosis of AA requires BM hypocellularity with at least 2 of thr following : HB < 10G/DL, ANC <1.5 X 10^9^//L, platelet count <50 x 10^9^//L and absolute reticulocyte count <60 x 10^9^//L [4]. In addition to this, there should be no abnormal cells, dysplasia or marrow fibrosis [6, 8–10]. Mrs KA fulfilled the diagnostic criteria and this classified her into having severe AA. In making the diagnosis other causes of pancytopenia such a s hypersplenism, severe megalobalstic anaemia BM infiltration, hypocellular acute leukemia, hypocellular myelodysplastic syndrome, myelofibrosis and PNH were ruled out from the clinical, laboratory and bone marrow findings [9]. The definite treatment of AA is with haematopoietic stem cell transplantation (HSCT) or immunotherapy using antithymocyte globulin (ATG) and cyclosporine. However, HSCT cannot cannot be done in pregnancy as the immunosuppressive therapy used are contraindicated in pregnancy [1–3, 6–8]. This makes management in pregnancy a big challenge. Although case reports have suggested a promising result with antithymocyte globulin (ATG) or cyclosporine therapy during pregnancy, there is currently little agreement on the universal use of these therapies [1, 2, 8, 11]. Guidelines from the British Committee for Standards in Haematology state that cyclosporine is safe in pregnancy but ATG, androgens or HSCT are not recommended [4]. Following delivery, cyclosporine was administered for this patient. Androgens were used before the advent of immunotherapy in AA but its use may cause the virilization of both the mother and female fetuses [10]. The efficacy of corticosteroids or granulocyte colony-stimulating factor is also equivocal [2, 3, 6, 10]. The use of high dose corticosteroid or granulocyte colony stimulating factor is also equivocal while majority of cases do not respond to the use of growth factors [6, 7, 10, 12]. Overall, current evidence does not favor the routine use of any drug therapy in the treatment of pregnancy-associated aplastic anemia [11]. However, when detected in early pregnancy (first trimester), most clinicians would offer a termination of pregnancy to enable definitive treatment to be initiated [3, 8–10].

Thus supportive therapy with serial red cell and platelet transfusions, with the use of prophylactic antimicrobials in neutropoenic patients is key [4]. The aim of supportive therapy is to carry pregnancy till term and delivery taken. Constraints in serial platelets transfusion for this patient were unavoidable due to the inability to provide platelet concentrate in this center. Blood donation and transfusion especially of blood components in this environment is less than optimal compared to developed countries. An attempt to transfer her to the nearest center in another region where this was available was rejected by the patient due to availability of family and financial support. This is a norm observed in this environment. All units of blood were donated by relatives who had their platelet count assessed in addition to routine pre-donation screening. All donors with platelet counts < 280 x 10^9^/L were rejected. In resource poor setting where platelet concentrates are unavailable, the use of fresh whole blood which is blood that is not refrigerated after collection and is transfused within an hour of donation may be used for thrombocytopenia. The mode of delivery is usually determined by obstetric reasons. Mrs KA had caesarean section because she developed severe hypertension and oligohydramnos and we were not comfortable with induction of labour having these two obstetric complications already. Though the relationship between AA and pregnancy is unclear, pregnancies associated with AA have been known to carry multiple maternal and fetal risks ranging from preterm birth, stillbirths, intrauterine growth restriction, miscarriages, severe preeclampsia, acute heart failure, gestational diabetes mellitus, and postpartum haemorrhage to puerperal sepsis [2, 3, 5, 6, 11]. Mortality from AA in pregnancy was between 20-60%, currently it is put at 2.7% due to improvement in supportive care [5, 6, 9, 10]. While a few cases go into complete remission estimated at about 25-30%, [11] follow up of these patients is vital as some of them relapse, which in pregnancy has been estimated at about 33% [9, 13]. Mrs KA is still on follow up and should remain on follow up in particularly in her subsequent pregnancies.

## Conclusion

Aplastic anaemia in pregnancy is a rare entity. It poses a lot of challenges to the obstetrician, haematologist and family. Supportive therapy with serial blood transfusion is key to successful pregnancy. Even in resource poor setting, fresh whole blood transfusions and joint management of AA in pregnancy is accompanied with satisfactory feto maternal outcomes.
